# Highlighting Shooting Opportunities in Football

**DOI:** 10.3390/s23094244

**Published:** 2023-04-24

**Authors:** Ilias Loutfi, Luis Ignacio Gómez-Jordana, Angel Ric, João Milho, Pedro Passos

**Affiliations:** 1 CIPER, Faculdade de Motricidade Humana, Universidade de Lisboa, Estrada da Costa, 1495-751 Cruz Quebrada, Portugal; loutfi.ilias@gmail.com (I.L.); luis.jordana.martin@gmail.com (L.I.G.-J.); 2 Complex Systems in Sport Research Group, Institut Nacional d’Educacio Fisica de Catalunya (INEFC), University of Lleida (UdL), 25192 Lleida, Spain; aric@gencat.cat; 3 CIMOSM, ISEL, Instituto Superior de Engenharia de Lisboa, Instituto Politécnico de Lisboa, 1959-007 Lisboa, Portugal; joao.milho@isel.pt

**Keywords:** high-tech video cameras, soccer, heatmaps, time and space, co-positioning, attacking patterns

## Abstract

The purpose of the present study was to create a two-dimensional model which illustrates a landscape of shooting opportunities at goal during a competitive football match. For that purpose, we analysed exemplar attacking subphases of each team when the ball was in the last 30 m of the field. The player’s positional data (x and y coordinates) and the ball were captured at 25 fps and processed to create heatmaps that illustrated the shooting opportunities that were available in the first and second half in different field areas. Moreover, the time that the shooting opportunities were available was estimated. Results show that in the observed match, most of the shooting opportunities lasted between 1 and 2 s, with only a few opportunities lasting more than 2 s. The shooting opportunities did not display a homogenous distribution over the field. The obtained heatmaps provide valuable and specific information about each team’s shooting opportunities, allowing the identification of the most vulnerable areas. Additionally, the amount, duration, and location of the shooting opportunities have shown significant differences between teams. This customizable model is sensitive to the features of shooting opportunities and can be used in real-time video analysis for individual and collective performance analysis.

## 1. Introduction

At present, positional data have become a usual tool for performance analysis in football. The main goal of a football match is to score a goal. With this purpose, several players are involved during every phase of the match, assuming different roles. The defending players are continually aiming to reduce the space of the opponents and, consequently, decrease the time available for the attacking squad to move the ball and create threatening situations (e.g., shot at goal), whereas the attacking players are continually exploring the space that was temporarily left available by the defenders to place the ball closer to the goal and as far as possible from the closest opponents or to shoot at the goal in an attempt to score. These paradoxical purposes create restricted possibilities of action for each team, for each moment of the match, and for each player. These opportunities of action can be conceptualized as affordances that emerge due to space–time constraints, which can be depicted by a landscape [1,2,3].

Affordances express an adaptive behaviour between an individual’s capacities and a particular set of properties of the environment [4,5,6]. Hence, it seems relevant to understand how the environment modulates the affordances of each player within a team. For instance, the ball carriers can be a temporary ‘leaders’, providing and creating key affordances to their teammates, orchestrating the offensive movements, and creating relevant information that helps their teammates to progress on the field or score a goal [7]. However, the behaviour of these ‘leaders’ simultaneously constrains and is constrained by the opponents’ and teammates’ behaviour. They are influenced by a reciprocal compensation that characterizes players co-adaptive behaviour in team sports [8,9,10].

Co-adaptation is a concept based on the perpetual adjustment of individuals to the changes induced by the adaptive actions of other individuals [11]. Within a competitive environment, co-adaptive behaviours are constrained by a complex combination of tasks and goals (some antagonistic) that characterize players’ interactive behaviours in space and time, supported by emergent local information [12]. This means that within a competitive match, the landscape of local information is continually changing, influencing players co-adaptive behaviours described by the dynamics of players’ relative positioning, players’ distances to the field boundaries, or the proximity to the scoring area [13,14,15,16].

Understanding the affordances that emerge during a match may enhance the capacity of a team to recognise the key affordances as well as the players who are able to shape correctly the movement pattern of a team in order to score a goal. Depicting a landscape of affordances (e.g., shooting opportunities) can also help to design specific training sessions to increase the acquisition of skills relative to these specific affordances [17,18].

Regarding the opportunities to score created during the course of a match, there is a gap in the literature in terms of depicting variations in shooting opportunities regarding the defending player’s motion. The traditional proxy used to evaluate players or team performance is generally the ratio of shots on goal or even the goal ratio. Neither of these proxies provide information about the qualitative aspect of the shooting opportunity, and they have a binary metric (goal or no goal) that limits the perception of the dangerousness [19] (or level of threat) of an opportunity. An exception could be the predictive model to estimate the goal scoring chance and the probability of scoring known as the expected goal (xG). This probability for each scoring chance, which ranges from 0 to 1, is calculated on the basis of event-based variables as: (i) shot location; (ii) distance to the goal; (iii) angle to the goal, calculated as the triangle between shot location and the goal posts; (iv) type of shot—part of the body used to score (i.e., feet, head, or other); (v) match sub-phase in which the goal was scored (e.g., open play, direct free-kick, corner kick, or counter-attack); and (vi) various scenarios regarding how a player controlled the ball before taking a shot (direct, volley, two touches, dribbling, or set-piece). Aimed to increase the predictive power of xG model, event data were combined with positional data [20]. On the basis of this, the goalkeeper positioning or the ‘pressure’ on the player who attempted to score were variables added to the model. The goalkeepers’ positional data were used to check whether they were in the line of shot, which was defined as a triangle between the shot location and the two goal posts. Positional data were also used to calculate the goalkeepers’ distance to the goal. Aiming to assess the level of threat by blocking the shot or the level of pressure over the shooter, the defenders’ positional data were used with this purpose. The number of defenders in the ‘line’ of shot (i.e., triangle between shot location and the goal posts) was counted to assess both the level of threat and the pressure over the shooter [20]. The defenders’ and goalkeepers’ positional data were captured only in the moment of the shot, which is acceptable for the stated purpose of feeding a machine learning algorithm. However, in this paper, we intended to demonstrate that positional data can be used beyond the quantification of the number of players within the line of shot, and we used players’ current and estimated positions to specify shooting opportunities that locally emerge due to players’ interactive behaviour during the course of a match [1,2].

Thus, it seems relevant to understand how specific spatio-temporal constraints (e.g., players co-positioning) influence the landscape of shooting opportunities during a competitive football match. Moreover, we hypothesized that these spatio-temporal constraints create a non-homogeneous distribution of the shooting opportunities, which allow for the identification of where and when the attacking squad creates more threatening situations for the defending team. Therefore, the main goal of this study was to elaborate a two-dimensional model that illustrates a landscape of shooting opportunities which emerge due to the interactive behaviours among the ball carrier, the closest defenders, and the goalkeeper during a competitive football match.

## 2. Materials and Methods

### 2.1. Data Acquisition

Data used in this study were captured from a recording of an official competitive football match from the Spanish second division during the season 2017/2018. All players were adult males. Each player position was described with bi-dimensional coordinates (x and y), and both axes were positioned at the level of the field boundaries, representing its length and width. The bi-dimensional coordinates were recorded at 25 fps and represent the main data used to test the capacity of our model to illustrate shooting opportunities. To reduce the noise associated to data collection with tracking systems, each player displacement was calculated with a frequency of 5 Hz.

Events such as passing, ball drives, fouls, exclusions, substitutions, goals, and kick off were defined to provide information allowing the recognition of the ball carrier.

At this stage of model development, simplicity was the rule, which means that other performance details were purposely left out. Similar to previous models regarding landscapes of opportunities of action, we decided to create this model adopting a top-view from the pitch. Thus, the 2D model for illustrating the landscape of shooting opportunities, presented here, was supported by each player’s bi-dimensional positional and events data. This reason supports our decision of not including other types of data, such as technical, tactical, game status (e.g., score), physiological, or psychological variables. These performance variables can be addressed in further stages of development of this model. The study was conducted in accordance with the Declaration of Helsinki, and the protocol was approved by the Ethics Committee of Faculdade de Motricidade Humana, Universidade de Lisboa, under the reference 25/2019. It analyzed performances that did not require identification of individual performers.

### 2.2. Procedures

First, we selected the plays by visual inspection, using as a criterion the entry of an offensive play within the last 30 m of the field. If the ball carrier or the ball went outside of these last 30 m towards the opponent goal, the offensive play was no longer considered. A total of 75 offensive plays were selected, 9 from the visitors’ team and 66 from the home team.

The second step consisted of defining the multiples events, which allowed our model to recognize the ball carrier at each moment of the match. The attacking team and the ball carrier were both defined using the events mentioned above and additional information over ball position. Based on the literature, we also set an average value of the ball speed during shooting between 25 to 35 m/s, with a mean value of 27.77 m/s or 100 km/h [21].

To increase the accuracy of our model, the defending players’ estimated positions (which allow or do not allow them to intercept a potential shot) was associated with the ability to change the running line direction. Previous research observed that the capacity to change direction is inversely related to the running speed on a straight line [22]. Grehaigne and colleagues defined a spherical area where a defender is capable to intervene while running. This area is decreasing in terms of space and in function of the defender’s actual speed. Thus, for each defending player, we defined a potential ‘sphere’ of action as a function of turning ability and speed. The highest turning ability (360°) was established for the lowest recordable speed (0 m/s) [1].

**Figure 1:**
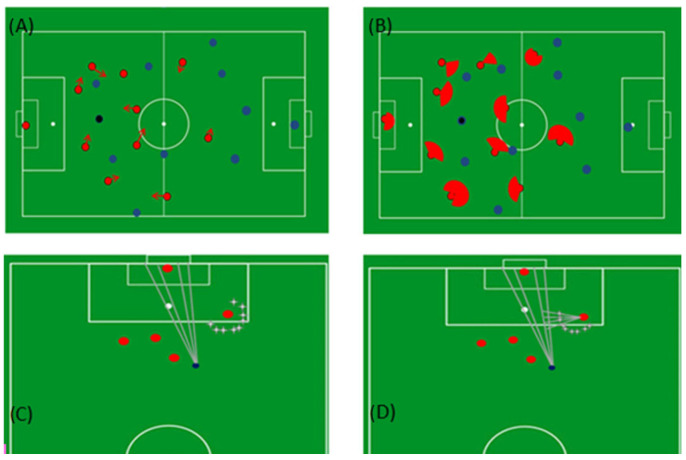
Schematic representations of the different steps used for modelling a shooting opportunity at goal. The blue dots represent the players of the attacking team, and the red dots represent the defending team. The ball carrier is represented with a black dot. (**A**) The red arrows represent the defender’s velocity vectors estimated by the algorithm. (**B**) The red spots represent the coverage area of the defending players. (**C**) The grey lines between the ball carrier and the goal represent hypothetical areas for shooting opportunities. (**D**) Grey line segments illustrate the estimated time a defender took to reach a hypothetical shooting opportunity. The white dots in (**A**,**B**) represent both penalty marks and the midfield. The white dots in (**C**,**D**) represent the penalty marks.

The potential sphere of action was defined as being the defensive coverage area of each defending player, corresponding to the space occupied even when their running speed is very low or null. The coverage area was defined using a trigonometric rule. First, we defined the angle (αp) in the field of the vector velocity of each defending player and for each position in time. Then, we considered *n* = 200 points (allowing the construction the coverage area, see Figure 1), that went through angles α_t_ + (α_p_/2) and αt-(α_p_/2) (Figure 1). Therefore, we can calculate each one of these points using:

𝑥_𝑛_ = 𝑥_𝑝_ (𝑡) +v_tp_ (𝑡) ∙ Δ𝑡 ∙ sin(𝛼_𝑛_) (1)

𝑦_𝑛_ = 𝑦_𝑝_ (𝑡) + v_tp_(𝑡) ∙ Δ𝑡 ∙ cos(𝛼_𝑛_) (2)

where *y_p_* and *x_p_* represent the position of a defending player at each moment of time, Δ𝑡 = 0.04 s is the time interval, v_tp_ is the total velocity of the defending player at each moment of time, and one of the 200 angles mentioned above is represented by α*_n_*. Finally, *x_n_* and *y*_n_ are the coordinates of the point of each of the *n* points of the coverage area [1]. For velocities below 1.5 m/s, the value of v_t_ was defined to be equivalent to 1.5 m/s, otherwise a defending player moving at a low or null speed will be considered and measured as covering no space. These vectors provide information regarding direction and velocity in a linear motion and allow our 2D model to recognize the intersection between the potential shooting trajectory and each defending player’s displacement vector.

### 2.3. Algorithm Description

To illustrate shooting opportunities, 20 separated lines starting from the ball carrier were created towards the goal (see Figure 1C,D). Furthermore, these hypothetical shooting lines create a reference for the time a defender will take to intercept them. If any defending player may intercept any of these hypothetical shooting lines, they were marked as a blocked shooting opportunity. The defenders’ ability to intercept or block a shooting opportunity was calculated through each defender’s coverage area (see Figure 1B,D).

**Figure 2:**
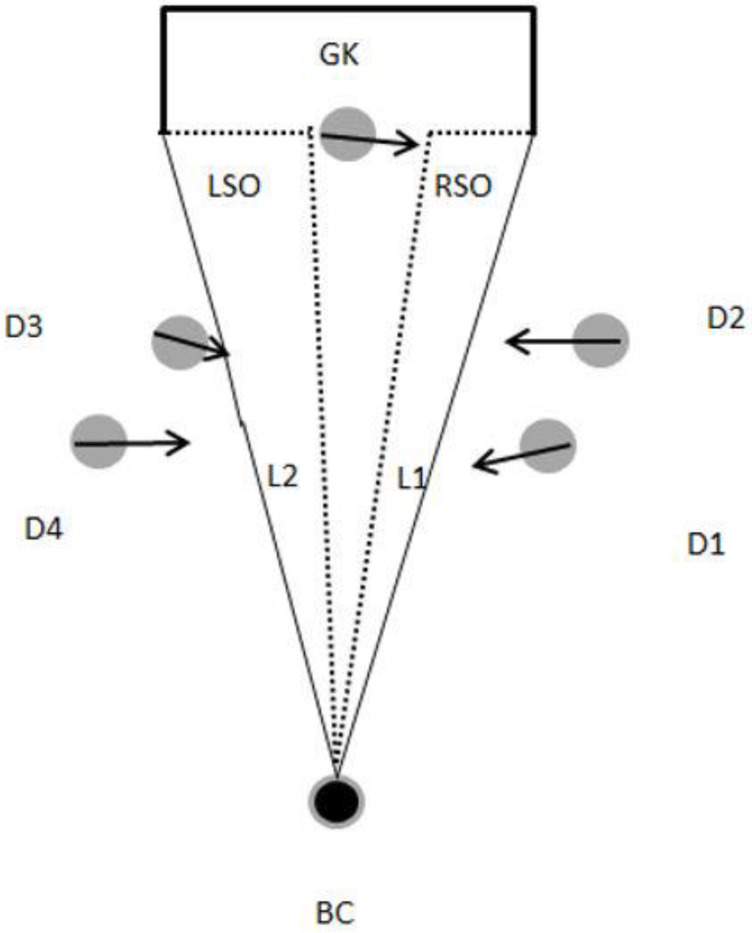
Polygon depiction of a shooting opportunity at goal. The grey circles represent the defending players (D1–D4) and the goalkeeper (GK). The black circle represents the ball carrier (BC). Each black arrow represents the player’s velocity vector. The black-dashed area with the black filled lines represents the shooting opportunities on the left side (LSO) and on the right side (RSO) of the goalkeeper.

As represented in Figure 1C,D, each shooting opportunity may occur on both sides of the goalkeeper or (dependent on the goalkeeper’s position) on one side only. Hence, to represent each shooting opportunity for the ball carrier, two polygons were created. One outlines the ball carrier, the goalkeeper’s current or estimated position, and the left post of the goal. Another polygon outlines the ball carrier, the goalkeeper’s current or estimated position, and the right post of the goal. The goalkeeper estimated position was calculated on the basis of the direction of the goalkeeper velocity vector (please see Figure 2). Each polygon allows the depiction of the space available for a shot to happen and was updated every 0.2 s, continuously assessing whether the defenders had the possibility to intercept or block a shooting opportunity.

The overlapping of the created polygons along the match allows the shaping of a heatmap that illustrates a bi-dimensional landscape of the shooting opportunities. The intensity of the colours (from dark blue to red) that were used to build the heatmaps helped to illustrate the superposition and the distribution of the obtained polygons (i.e., the shooting opportunities) in space.

### 2.4. Distribution of the Shooting Zones and the Level of Threat

**Figure 3:**
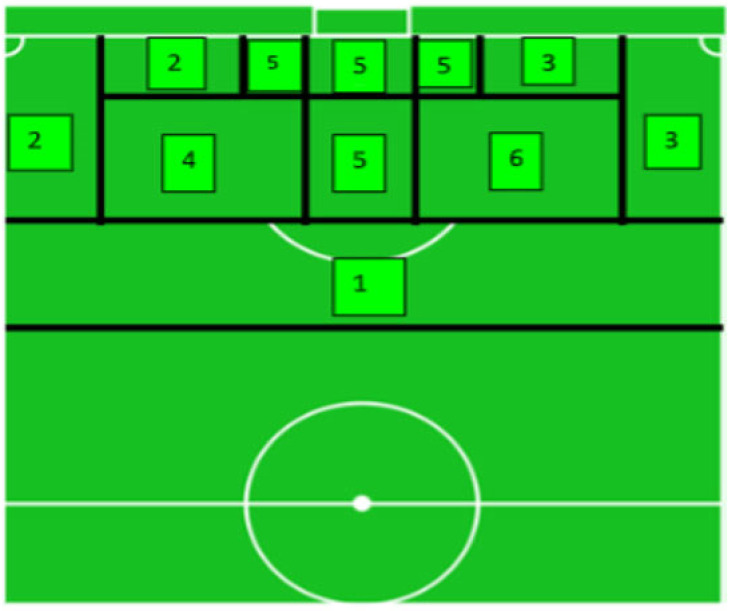
Distribution of the shooting zones in the last 30 m inspired by the work of Rathke (2017). We chose to divide the zones in function of the level of threat they represent. The most threatening zones associate an optimal angle of shot with a favourable distance to the goal, such as Zones 4, 5, and 6. The eccentric or lateral zones (Zones 2 and 3) represented a lower threat level. Finally, the zone located outside of ‘the box’ (Zone 1) is characterized by a greater distance to the goal and, therefore, represents a medium level of threat.

According to the area of the field, shooting opportunities may vary the level of threat for the defending team. Thus, we decided to divide the last 30 m of the pitch into 6 zones with different sizes and shapes (please see Figure 3). This division was made as a function of the penalty area (usually called ‘the box’), which is a rectangular area that extends 16.5 m to each side of the goal (33 m in total) and 16.5 m in front of it. The literature shows evidence that there is a statistical difference in goals scored between shots inside ‘the box’ and outside of ‘the box’ [23]. Additionally, [24,25] identified (i) zones where teams attempted more shots; (ii) zones with more shots towards the goal; and (iii) zones where a greater number of goals were scored. These zones are a simple extension of ‘the box’ line boundaries and an additional line, which marked the 30 m of distance to the goal [26]. This zone representation provides an accurate identification of the origin of the shooting opportunities and enriches the spatial information obtained from the heatmaps.

### 2.5. Statistical Procedures

For a better understanding of the dispersion of the results concerning the time that shooting opportunities were available, the following descriptive statistics were calculated for both teams: (i) the mean and the standard deviation of the time that the shooting opportunities were available; (ii) the maximum and the minimum time that shooting opportunities were available; (iii) the quartiles; and (iv) the interquartile range.

Then, the sample (*n* = 75) was tested to see whether it has a normal distribution. The Shapiro–Wilk and Kolmogorov–Smirnov tests of normality identified that the time that the shooting opportunities were available had a non-normal distribution. Since the normality test showed that results did not have a normal distribution, it was decided to use a non-parametric tests to compare the results of the non-normally distributed sample. Thus, with the help of the software IBM SPSS Statistics, a non-parametric test was applied to compare the time that the shooting opportunities lasted for both teams, as well as regarding the zone location of those shooting opportunities. The independent-sample Kruskal–Wallis test with a *p*-value of 0.05 was used for this purpose.

## 3. Results

### 3.1. Landscapes of Shooting Opportunities

A heatmap was built for each one of the teams, representing the landscapes of shooting opportunities for the first and the second half separately. For each shooting opportunity, the origin of the shot was detected using the zone’s distribution mentioned above; two heatmaps for shooting opportunities inside and outside ‘the box’ were represented separately. Concerning the model’s validity, the results unravel that for the more than 27 shoots that occurred during this match, the algorithm detected 70.3% of them. This result represents a good ratio of detected shots, and it seems that this 2D model was able to recognize the shooting opportunities available for a certain amount of time. It is clear that depicting an affordance of shooting opportunity does not necessarily mean depicting an effective shot. These type of affordances can provide contextual information to explain players’ behaviour beyond the information of shooting or not shooting.

#### 3.1.1. Heatmaps of the Home Team

**Figure 4:**
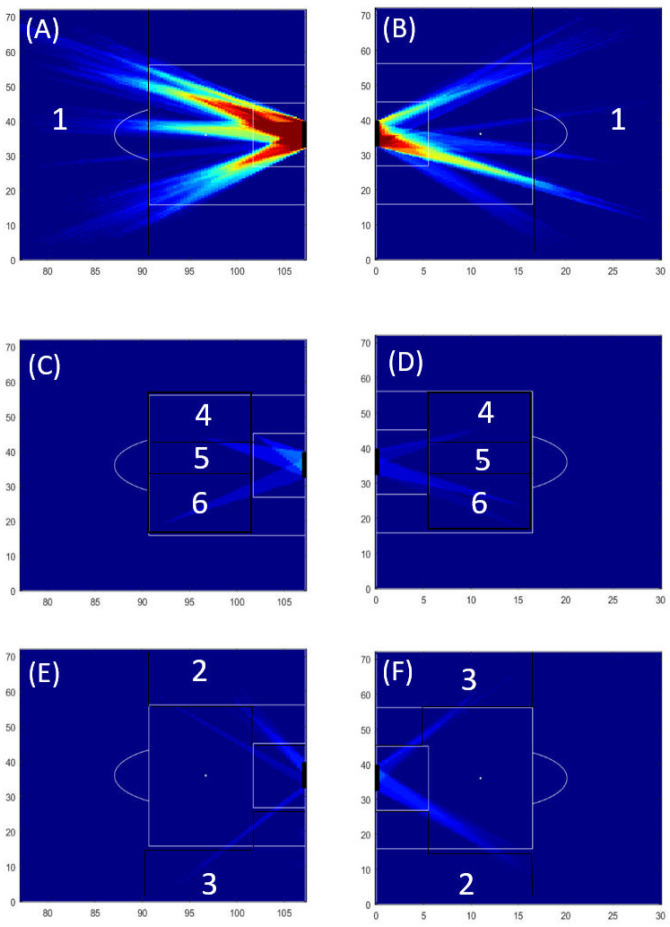
Heatmaps of the shooting opportunities of the home team. The heatmaps (**A**,**B**) illustrate shooting opportunities created outside ‘the box’ (Zone 1) for the first and second half, respectively. The heatmaps (**C**,**D**) illustrate the lateral shooting opportunities (Zones 2 and 3) for the first half and second half, respectively. Finally, the heatmaps (**E**,**F**) illustrate the shooting opportunities inside ‘the box’ (Zones 4, 5, and 6) for the first half and second half, respectively. A colour scale was used to provide information of the frequency of the shooting opportunities. Dark blue means that no shooting opportunity was available, whereas dark red means that shooting opportunities were available for more than 100 s per match.

Our results illustrated that for both halves of the match, most of the shooting opportunities of the home team were created in Zone 1 (please see Figure 4A,B). Fewer shooting opportunities were created in Zones 4 and 6 and no shooting opportunities were created in Zone 5 (Figure 4C,D). In addition, fewer shooting opportunities were created from the lateral zones (Zones 2 and 3) of the field (please see Figure 4E,F). 

#### 3.1.2. Heatmaps of the Visitors’ Team

**Figure 5:**
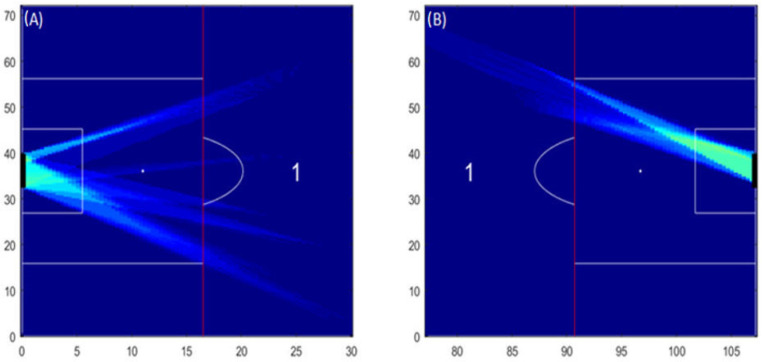
Heatmaps of shooting opportunities of the visitors’ team. The heatmaps (**A**,**B**) illustrate shooting opportunities created outside ‘the box’ (Zone 1) for the first and second half, respectively. No other shooting opportunities were depicted by the algorithm in the remaining zones (i.e., the areas inside ‘the box’ and the lateral ones—on the second half). A colour scale was used to provide information of the frequency of the shooting opportunities. Dark blue means that no shooting opportunity was available, whereas dark red means that shooting opportunities were available for more than 100 s per match.

Our results illustrated that for both halves of the match, most of the shooting opportunities of the visitors’ team were created in Zone 1 (please see Figure 5A,B). Moreover on the second half of the match the visitor team can only created shooting opportunities on the right side of zone 1 (Figure 5B). No shooting opportunities were created for the remaining zones.

### 3.2. Descriptive Analysis of Shooting Opportunities

The model estimated 75 shooting opportunities for both teams. The descriptive statistics concerning the duration of the shooting opportunities are detailed in Table 1. We can observe that an opportunity of shot at goal during this match was available, on average, for 1.37 s ± 0.82 s. The mean time available for a shooting opportunity was higher for the visitors’ team (1.6 s ± 1.1 s) than for the home team (1.34 s ± 0.78 s). We can also observe the dispersion of the time of each shooting opportunity with the interquartile range; it contains the first and third quartile of the sample and allows us to observe that the data are not very scattered but clustered around the median.

The spatial locations of the shooting opportunities are detailed in Table 2. Considering both teams, approximately 85% of the shooting opportunities were located in Zone 1 (i.e., outside ‘the box’). The home team was able to create more shooting opportunities, especially in Zone 1; concerning the most threatening zones, the home team created only 3% of the shooting opportunities in Zones 4 and 6, whereas the visitors’ team was able to create shooting opportunities only in Zone 1. Neither of the teams was able to create shooting opportunities in Zone 5.

Concerning the zones where the shooting opportunities of the home team were created, the opportunities did not last the same amount of time. As displayed in Table 3, Zone 3 was the area of the field were the shooting opportunities for the home team lasted a shorter time, whereas Zone 2 was the area of the pitch where the shooting opportunities were available for a longer time.

### 3.3. Statistical Test of Shooting Opportunities

There were no significant differences concerning the time that shooting opportunities were available between both the home team H(65) = 66, *p* = 0.477 and the visitors’ team H(7) = 7, *p* = 0.429. In addition, no significant difference was found for the time availability of shooting opportunities regarding the zone location of the shooting opportunities for either the home team H(65) = 65, *p* = 0.477 or the visitors’ team H(7) = 7, *p* = 0.429.

## 4. Discussion

### 4.1. Heatmaps and the Threatening Zones

The visual inspection of the heatmaps allows us to characterize the different zones of the field where shooting opportunities were created. Similar to previous research which illustrates landscapes of passing opportunities [1], our model also reinforces the notion that players’ opportunities of shooting were relative to their co-positioning. These results consolidate the idea that threatening situations (such as shooting opportunities) in the course of a competitive football match are quite dependent on the interactive behaviour among opponent players, at least in the last 30 m of the field.

These results can also be an indicator of the hegemony of a team, also called dominance [19]. A dominant team can be theoretically characterized by its ability to explore the space that was temporarily left available by the opponents, which consequently can lead to the creation of threatening situations. Our results illustrated that the home team was more dominant in terms of shooting opportunities created during this match, especially in crucial zones (the literature defines crucial zones in terms of shots/goal ratio or goals) as Zones 4 and 6 [25]. In addition, we can suggest that for the attacking team, the less threatening areas can allow for more shooting opportunities, since the defending team is more prone to cover vital spaces, such as those located inside ‘the box’.

Our results confirmed that Zone 5 displayed fewer shooting opportunities than the other zones, suggesting that the defenders were making a considerable effort to be well positioned in this zone and succeed in avoiding the emergence of any shooting opportunity in front of the goal. As a consequence, no shooting opportunities were created from Zone 5, indicating that neither team succeeded in creating short-range opportunities. However, despite the few opportunities identified by the model, many shots were performed, which suggest that closest to the goal, attackers use many opportunities to take a shot at the goal, even if the path towards the goal was filled by opponent defenders in position to neutralize these potential threats. Creating only a few (or no!) shooting opportunities inside ‘the box’ may provide information about the inability of a team to unbalance the defensive squad in the last third of the field. A situation that may be due to a lack of ability to create space that affords a shot inside ‘the box’.

For the match under analysis, our results highlight that the central position of the goal is indeed the most threatening zone. The defending players are continually positioning in Zones 4, 5, and 6, annihilating all the potential shooting opportunities inside ‘the box’, intentionally leaving the outside of ‘the box’ (Zones, 1, 2 and 3) to be eventually explored by the opponent (to pass or cross the ball into the area). This could be a main reason for why both teams created the highest number of shooting opportunities outside ‘the box’ (Zone 1 in Figure 1B). The greater distance to the goal causes defenders to perceive this area as less threatening. Consequently, more space is left available for longer by the defenders, which makes that area outside of ‘the box’ prolific for key passes—with emergent opportunities for penetrative passes to place the ball on a support player closest the goal, not to use that space and instead keep the ball in order to attract opponent defenders and create space in their back, or at last, offering an affordable distance for a long shot [24]. Obviously, these results could not be generalized, but they reveal the ability of our model to unravel the most threatening zones that may emerge during the course of a football match. Generalization associated with the frequency, location, and time length of shooting opportunities demand a larger sample of competitive matches, which is not the purpose of this study at this stage of the model development. Moreover, some caution is necessary in interpreting these results since they are based on analysis of a 2D model of a landscape, and their generalization, to some extent, is limited.

### 4.2. The Length of the Shooting Opportunities

The defending players are continually managing the space between them, aiming to annihilate or block any possible shot trajectory. Consequently, the created shooting opportunities were available only for a short time window. This emergent task constraint, also seen as the ‘pressure’ applied over the shooter, may influence the location of the shooting opportunity, the type of finishing (volley, finishing on the ground, etc.), or the shooting accuracy [27]. Due to the quantity of the shooting opportunities outside ‘the box’ (85.3% of all shooting opportunities created by both teams) and the time that these opportunities lasted (in comparison with other zones), it is hypothetically possible that the creation of a shooting opportunity (in front of ‘the box’, for example) may be valuable for further progression towards the goal, allowing a better shooting angle or a shot from a closer distance.

We can notice that the home team was able to create shooting opportunities in more threatening zones, especially Zones 4 and 6 (i.e., inside ‘the box’). The spatial location, as well as time availability of these shooting opportunities, may suggest that the home team was more efficient and successful in creating shooting opportunities in various positions in the field. On the contrary the visitors’ team created opportunities only in Zone 1 (i.e., outside ‘the box’) and was not able to create shooting opportunities in different zones or within a closer range. However, this team created the shooting opportunities that lasted longer, with a mean time of 1.6 ± 1.1 s slightly longer than their opponent.

The time availability can be considered as a qualitative factor allowing for the estimation or evaluation of the dangerousness of a shooting opportunity [28]. Our results illustrate that the major length of time that shooting opportunities lasted (in this match) was in a time window from 1.0 s to 2.0 s. In relative values, this means that 45.3% of the total amount of shooting opportunities lasted between 1.0 and 2.0 s, only 20% of the shooting opportunities lasted more than 2 s, and 34.6% of the created shooting opportunities lasted less than 1 s. Notwithstanding the lack in the scientific literature concerning the time availability of shooting opportunities—which makes it difficult to compare those values with other references—this reinforces the novelty that this model brings to the literature.

Similar to previous research, this is a methodology-driven research paper [1]. Therefore, we do not aim to generalize the results but rather aim to provide a generalization of the method applicability. Nevertheless, the space–time constraints associated with the shooting opportunities on each zone provide useful information for practice designs. Manipulating these space–time constraints, for instance, by increasing or decreasing the attacker–defender interpersonal distances on each trial, create practice conditions for the improvement of the accuracy of perception and action couplings associated with the shooting opportunities that are (or are not!) available.

### 4.3. Limitations of the Model and Issues for Further Research

Some limitations were observed during the construction of this model. The first is concerned with the bi-dimensionality of our model; despite x and y coordinates revealing enough to quantify and illustrate a landscape of shooting opportunities, it underestimates the complexity of a football match, since football is an activity that unfolds on a three-dimensional space. This creates some limitations to accurately assess the goalkeeper’s actions to defend a shot on goal, since shots can go towards the upper part of the goal. The literature showed evidence that for approximately 53% of the scored goals during a season in professional football matches, the ball went below the height level of the goalkeeper’s ankle [29]. Moreover, we are considering the defenders potential to intercept a shot only at the ground level. We are aware that up to a certain distance from the defender, the ball carrier may have a shooting opportunity for the ball to go upward towards the highest part of the goal. These arguments emphasize the relevance of the third dimension, which we intentionally left out at this stage of development of the model but which is noted as an issue for further research. Finally, close to the goal, the ball carrier has to make the decision of shooting or passing to a teammate located in a more threatening zone, which increases the chances of scoring. It is possible to hypothesize that if the opportunity to perform a penetrative pass to a support player it is not available, then the ball carrier may decide to shoot. Thus, the ball carrier’s teammates’ relative positions are variables that could be considered in the next stages of this model.

## 5. Conclusions

We may conclude that two-dimensional positional data used to describe players’ interactive behaviour of both teams form a suitable basis to create the heatmaps that illustrate the landscapes of shooting opportunities.

This 2D model can be extended to other levels of performance and to other team sports where space–time constraints bound players’ opportunities of action.

The uniqueness of each heatmap highlights the specificity of the space–time constraints which drive players’ behaviour during the course of a match.

Our 2D model illustrated the shooting opportunities that occur during a football match and provided crucial information over spatial location and time availability of those opportunities. The spatial locations and time lengths of these shooting opportunities were not homogenous over the field; on the contrary, some zones were overused in comparison with others, and the time of their availability was not similar. As expected, the less threatening zones (located far from the goal, with the widest angle to the goal) were areas where more shooting opportunities were created, whereas zones located closest and more in front of the goal, usually considered as high threatening zones, were those with few or no shooting opportunities.

Finally, we may also conclude that the results of this shooting opportunity model provide information that could be appropriate to a post-match report shortening the gap between performance analysts and the coaches’ technical staff.

## Figures and Tables

**Table 1:** Descriptive statistics of shooting opportunity duration (s).

	Number of Shooting Opportunities	Mean Time (s) and Standard Deviation (s)	Median Time (s)	Max Time (s)	Min Time (s)	1st Quartile (s)	3rd Quartile (s)	IQ Range (s)
Home Team	66	1.34 ± 0.78	1.2	3.6	0.4	0.6	1.8	1.2
Visitors’ Team	9	1.6 ± 1.1	1	3.2	0.4	0.8	2.9	2.1
Total	75	1.37 ± 0.82	1.2	3.6	0.4	0.6	1.8	1.2

**Table 2:** Percentage of spatial location of shooting opportunities.

	Zone 1	Zone 2	Zone 3	Zone 4	Zone 5	Zone 6
Home team	83%	3%	7.5%	3%	0%	3%
Visitors’ team	100%	0%	0%	0%	0%	0%
Total	85.3%	2.6%	6%	2.6%	0%	2.6%

**Table 3:** Average time (s) and standard deviation of shooting opportunities per zone.

	Zone 1	Zone 2	Zone 3	Zone 4	Zone 5	Zone 6
Home team	1.4 ± 0.1	1.5 ± 0.1	0.68 ± 0.13	1.3 ± 0.1	---	1.2 ± 0.4
Visitor’s team	1.6 ± 1.1	---	---	---	---	---

## Data Availability

The data presented in this study as well as the code created to generate the algorithm will be available on request from the corresponding author. Moreover, if the paper is accepted for publication, we commit to add the data and the code to a public repository.
